# Supplementary material for: Bacterial origins of thymidylate metabolism in Asgard archaea and Eukarya
Source: Nat Commun. 2023 Feb 15;14:838. doi: 10.1038/s41467-023-36487-z (PMC9931769; doi:10.1038/s41467-023-36487-z)

List of supplementary figures:

1. Gene neighborhood of *Psyn* thyX with accession numbers
2. List of *Psyn* folate dependent enzymes
3. Annotated HGTector analyses of predicted gene transfer events in *Psyn*, including the distribution of close and distal scores
4. Phylogenetic tree of ThyX (annotated)
5. Phylogenetic tree of ThyA (annotated)
6. Phylogenetic trees of ThyX and ThyA (with high confidence branches)
7. Phylogenetic tree of FTHFS
8. Phylogenetic tree of FolA
9. Phylogenetic tree of PurH
10. Phylogenetic tree of MTHFS
11. Phylogenetic tree of MTHFR
12. Phylogenetic tree of MetH
13. Phylogenetic tree of FolD
14. Reconciliation analysis ThyX
15. Reconciliation analysis ThyA

Supplementary Figure 1: Gene neighborhood of *Psyn thyX* with accession numbers. % of archaeal and Asgard top hits is also indicated.

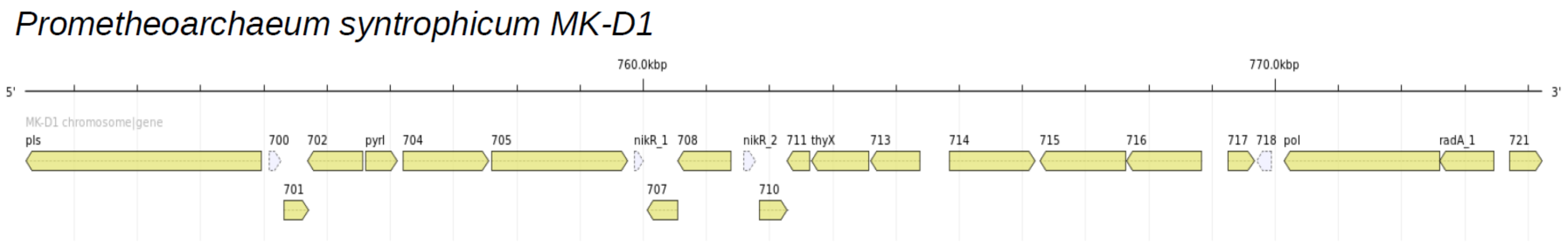

Gene	First BLASTP Hit	E-value	% Archea Top100 Hit	% Asgard Top100 Hit
pls	Candidatus Lokiarchaeota archaeon	0.0	43	42
701	Candidatus Lokiarchaeota archaeon	2E-33	100	97
702	Candidatus Lokiarchaeota archaeon	6E-12	95	57
PyrL	Candidatus Lokiarchaeota archaeon	9E-74	100	97
704	Candidatus Lokiarchaeota archaeon	2E-32	11	8
705	Candidatus Lokiarchaeota archaeon	0.0	100	47
706	Candidatus Lokiarchaeota archaeon	1E-19	100	97
707	Candidatus Lokiarchaeota archaeon	2E-39	100	98
nikR	Candidatus Lokiarchaeota archaeon	2E-19	100	53
710	Candidatus Methanofastidiosa archaeon	7E-64	100	94
711	Thermoprotei archaeon	6E-21	100	21
thyX	Zixibacteria bacterium	2E-78	3	2
713	Lokiarchaeum sp. GC14_75	3E-43	94	94
714	Lokiarchaeum sp. GC14_75	1E-56	92	90
716	Nitrospiraceae bacterium	4E-87	34	11
717	Candidatus Lokiarchaeota archaeon	1E-47	87	44
pol	Candidatus Lokiarchaeota archaeon	0.0	77	47
radA	Candidatus Lokiarchaeota archaeon	5E-53	81	72
721	Candidatus Helarchaeota archaeon	1E-45	39	12

Supplementary Figure 2

Psyn folate dependent of enzymes with their accession numbers and protein/gene names following a bacterial nomenclature

ID	Name Protein, gene	Activity	Evidence (HHPRED)		Expression	
			Prop (%)	e-value	This work	Original analysis
WP_147662173.1	DHFR, folA	Dihydrofolate reductase	99.4	10^{-24}	254.85	210
WP_147661168.1	FTHFS, fhs	formate--tetrahydrofolate ligase	100	10^{-201}	293.96	269
WP_147664305.1	FolD, folD	bifunctional 5,10-methylenetetrahydrofolate dehydrogenase/5,10-methenyltetrahydrofolate cyclohydrolase	100	10^{-44}	216.68	215
WP_147661599.1	MetF, metF	methylenetetrahydrofolate reductase [NAD(P)H]	100	10^{-49}	250.91	258
WP_147665170.1	MetH, metH	Methionine synthase	100	10^{-76}	267.28	253
WP_147662661.1	SHMT, glyA	Serine hydroxymethyl transferase	100	10^{-35}	379.69	318
WP_147661826.1	ThyX, thyX	Thymidylate synthase (FAD)	100	10^{-52}	225.37	227
WP_147664706.1	PurH, purH	bifunctional phosphoribosylaminoimidazolecarboxamide formyltransferase/IMP cyclohydrolase	100	10^{-121}	78.97	57
WP_147661605.1	GcvT, gcvT	glycine cleavage system aminomethyltransferase	100	10^{-53}	279.69	257
WP_162306824.1	MTHFS, ygfA	5-formyltetrahydrofolate cyclo-ligase	100	10^{-46}	101.87	63
WP_147661922.1	FTCD, ftcD	Glutamate formimino transferase	100	10^{-121}	329.56	317
WP_147661705.1	PanB	3-methyl-2-oxobutanoate hydroxymethyltransferase	100	10^{-59}	880.24	764

Supplementary Figure 3: Raw data from the HGTector analysis refering to Fig. 3 of the manuscript. Distributions of close and distal scores are also indicated.

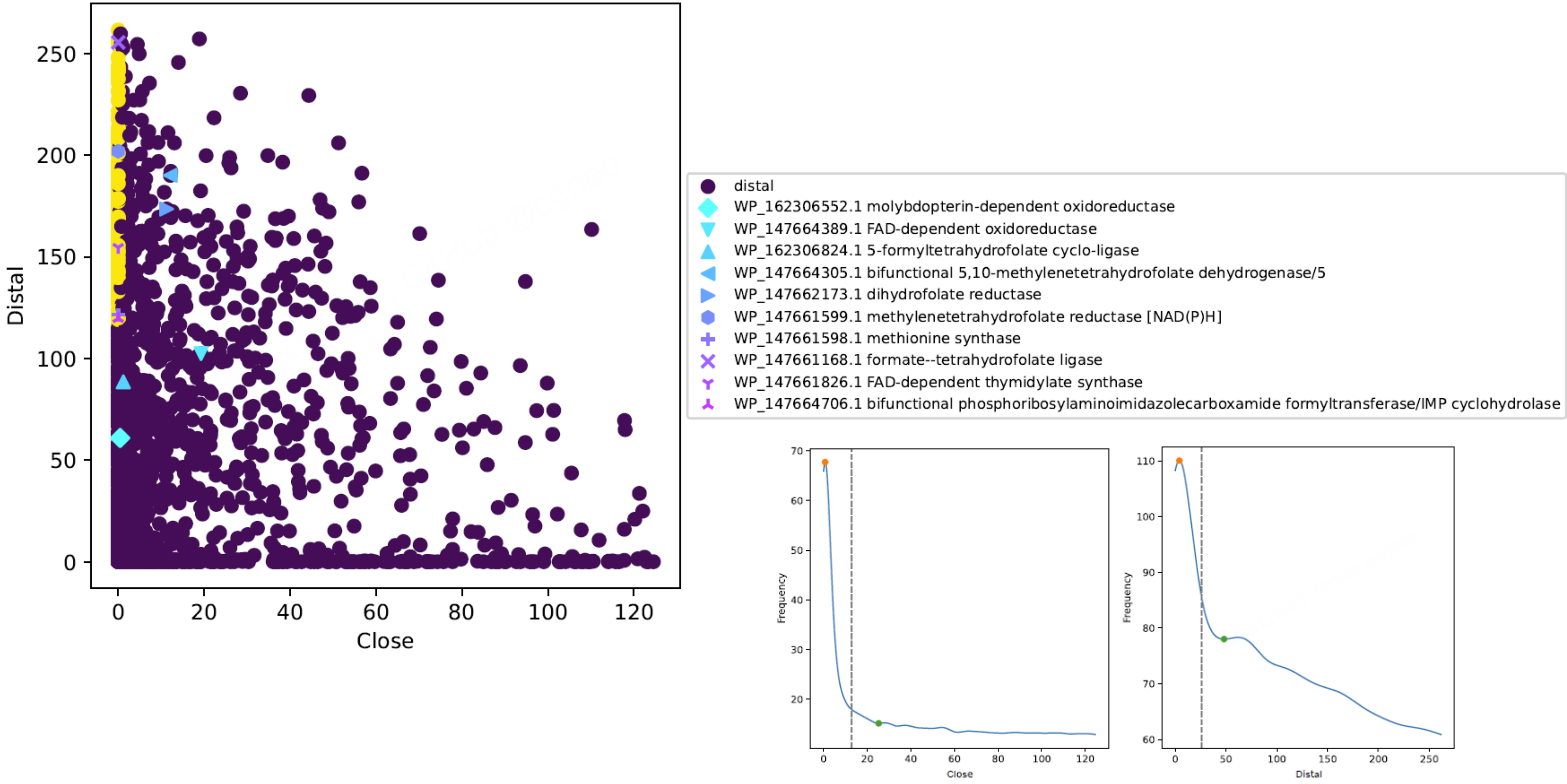

Distributions of close and distal scores. The dotted line indicates automatically determined thresholds by the HGTector.

Supplementary Figure 4: Non-rooted IQ-TREE of ThyX with bootstrap values.

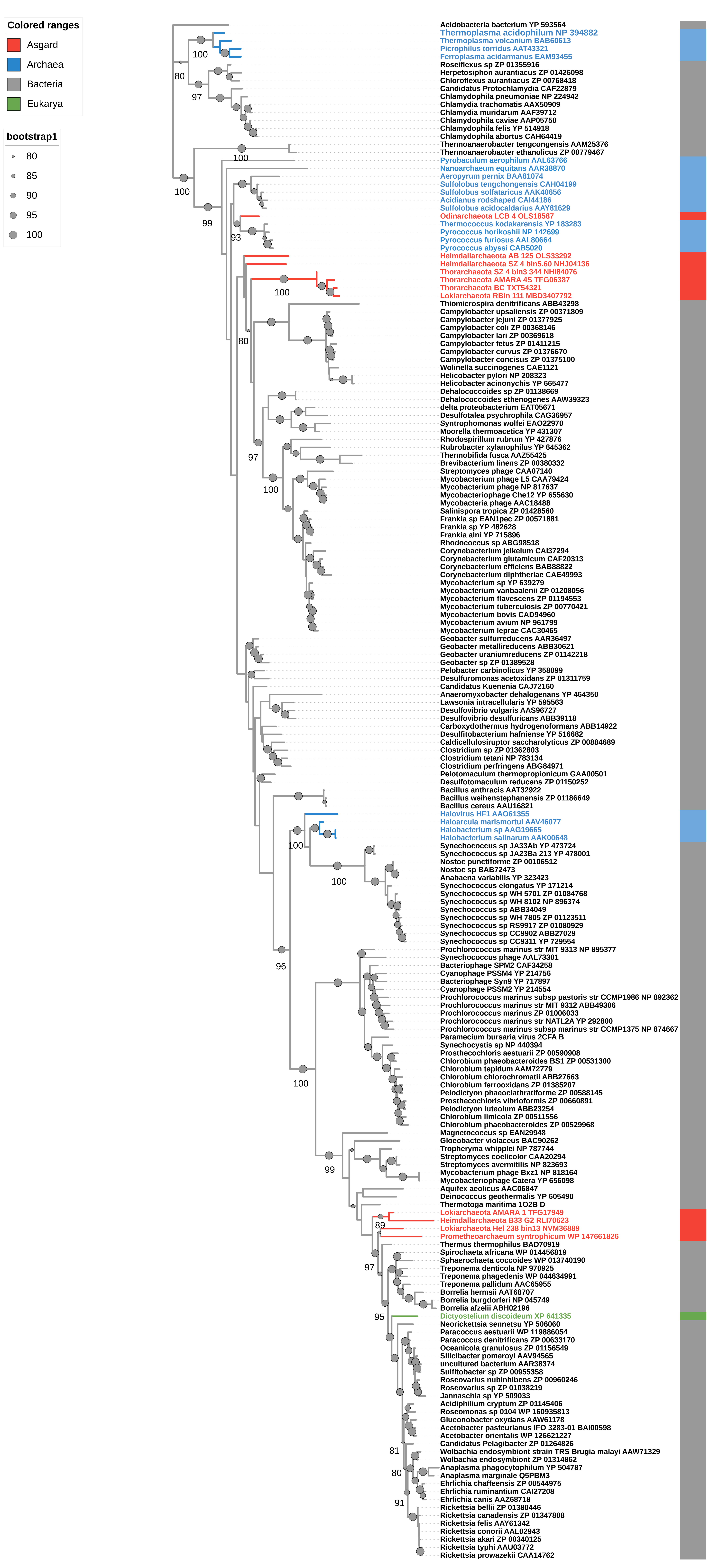

Supplementary Figure 5: Non-rooted IQ-TREE of ThyA with bootstrap values.

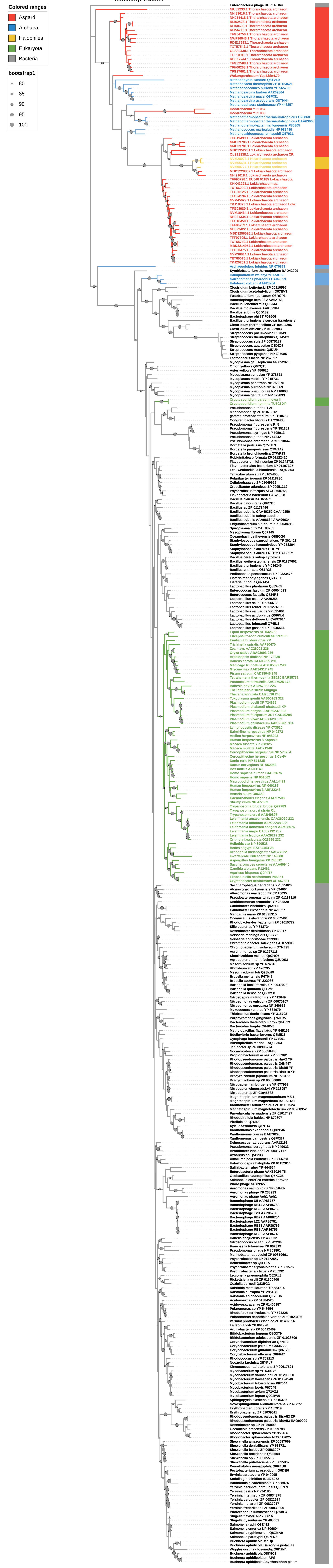

Supplementary Figure 6: High confidence ThyA and ThyX phylogenetic trees.

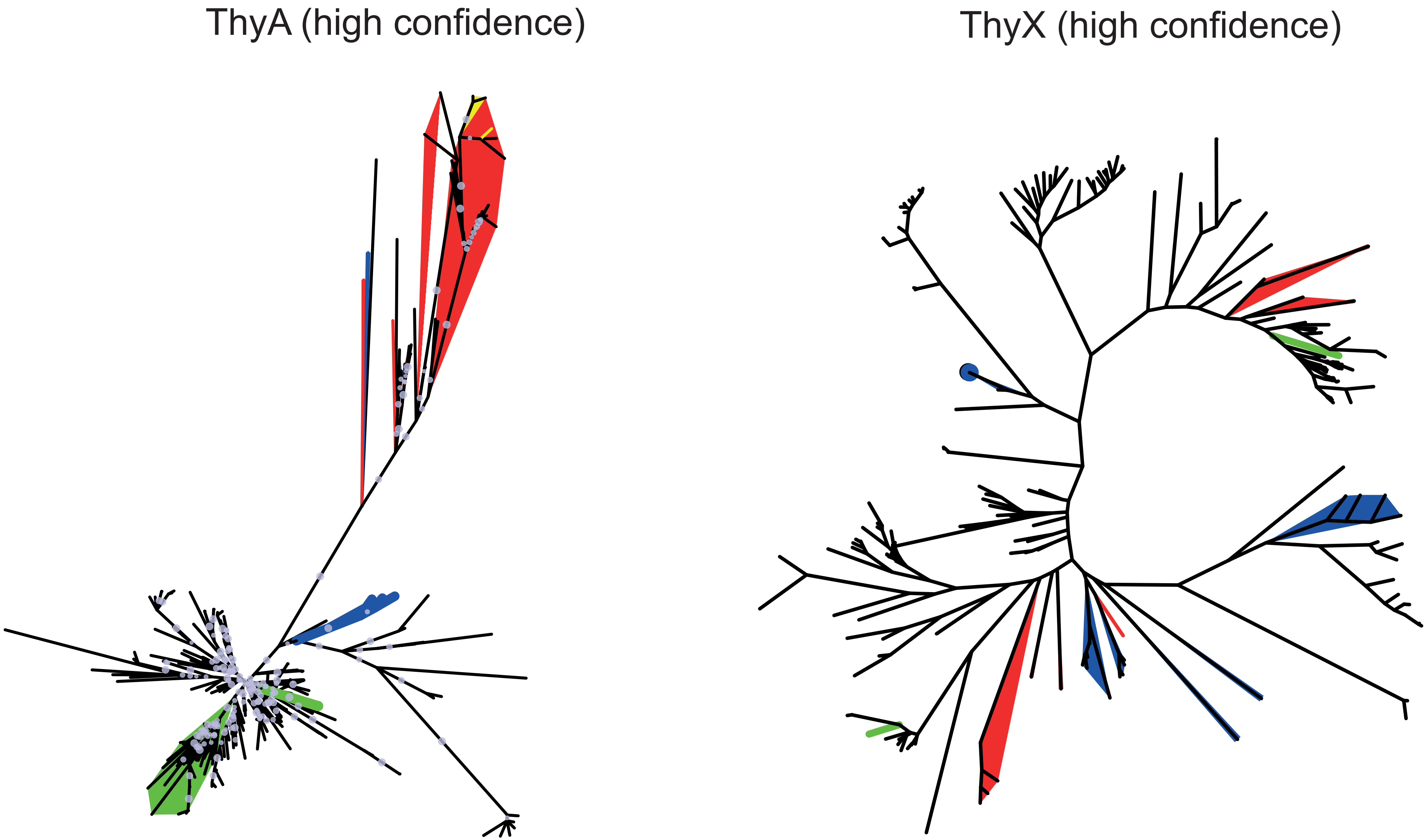

Refers to Fig. 4, branches with bootstrap support less than 85 were deleted

Supplementary Figure 7: Non-rooted IQ-TREE of FTHFS with bootstrap values.

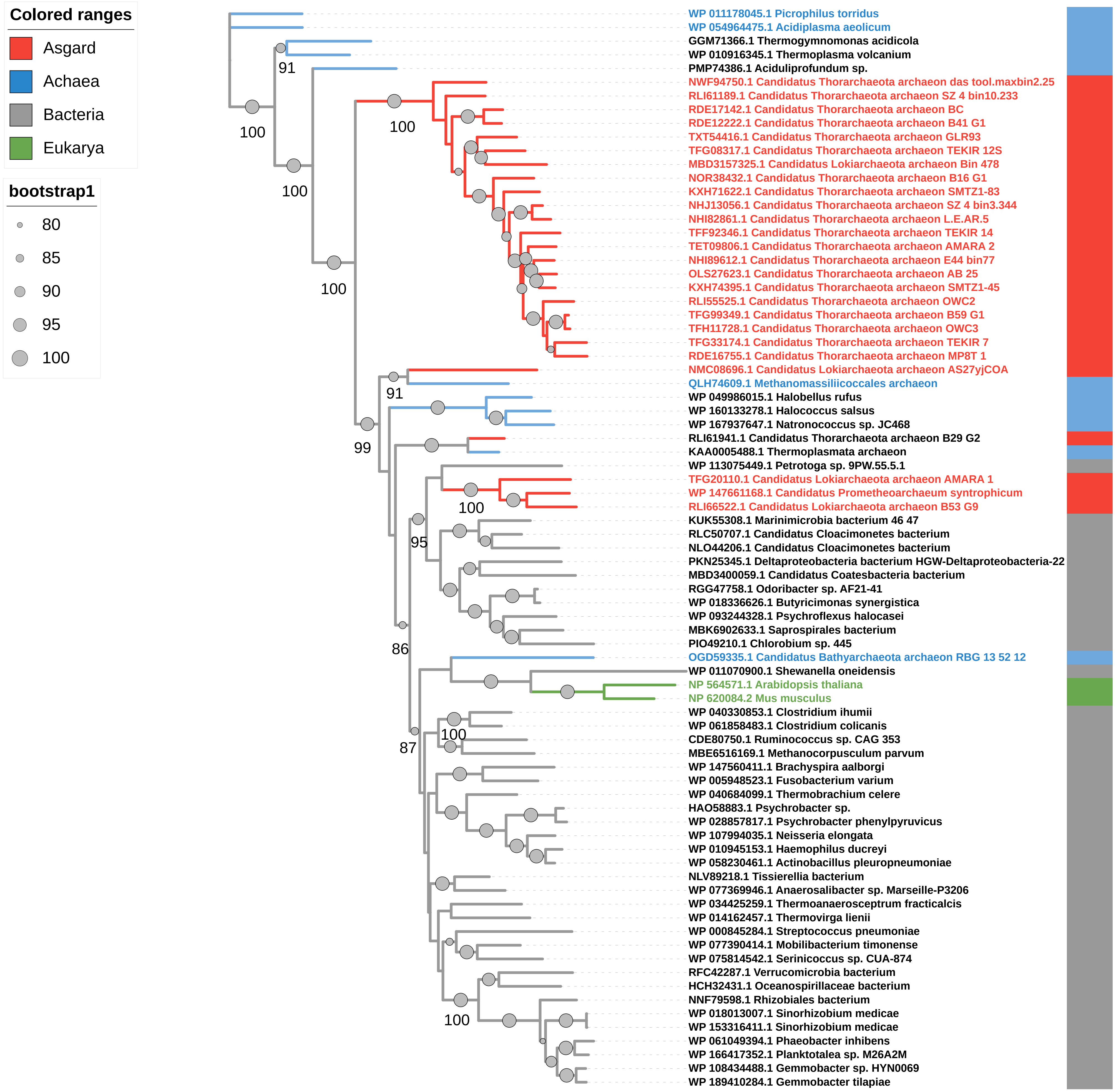

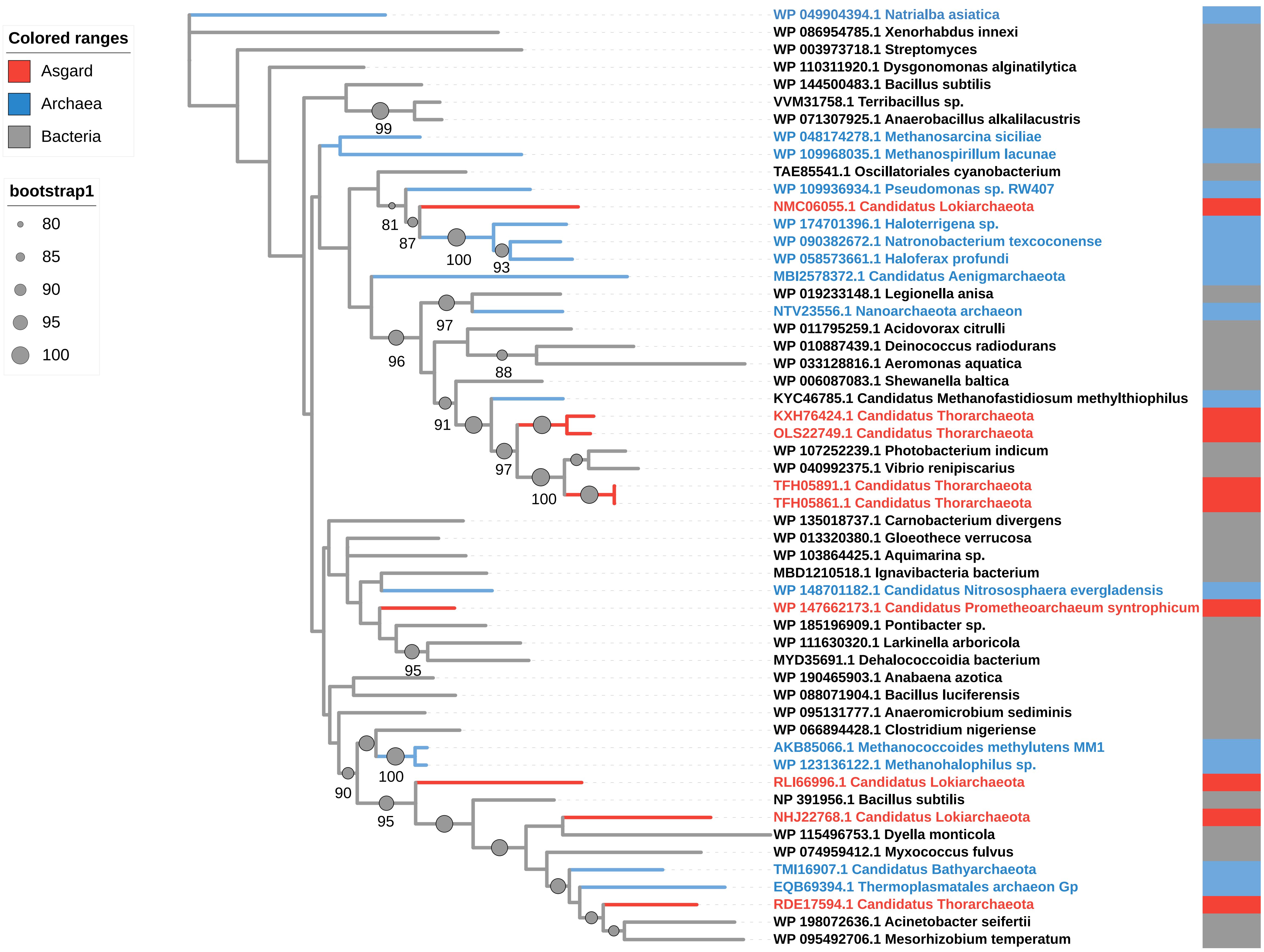

Supplementary Figure 8: Non-rooted IQ-TREE of FolA with bootstrap values.

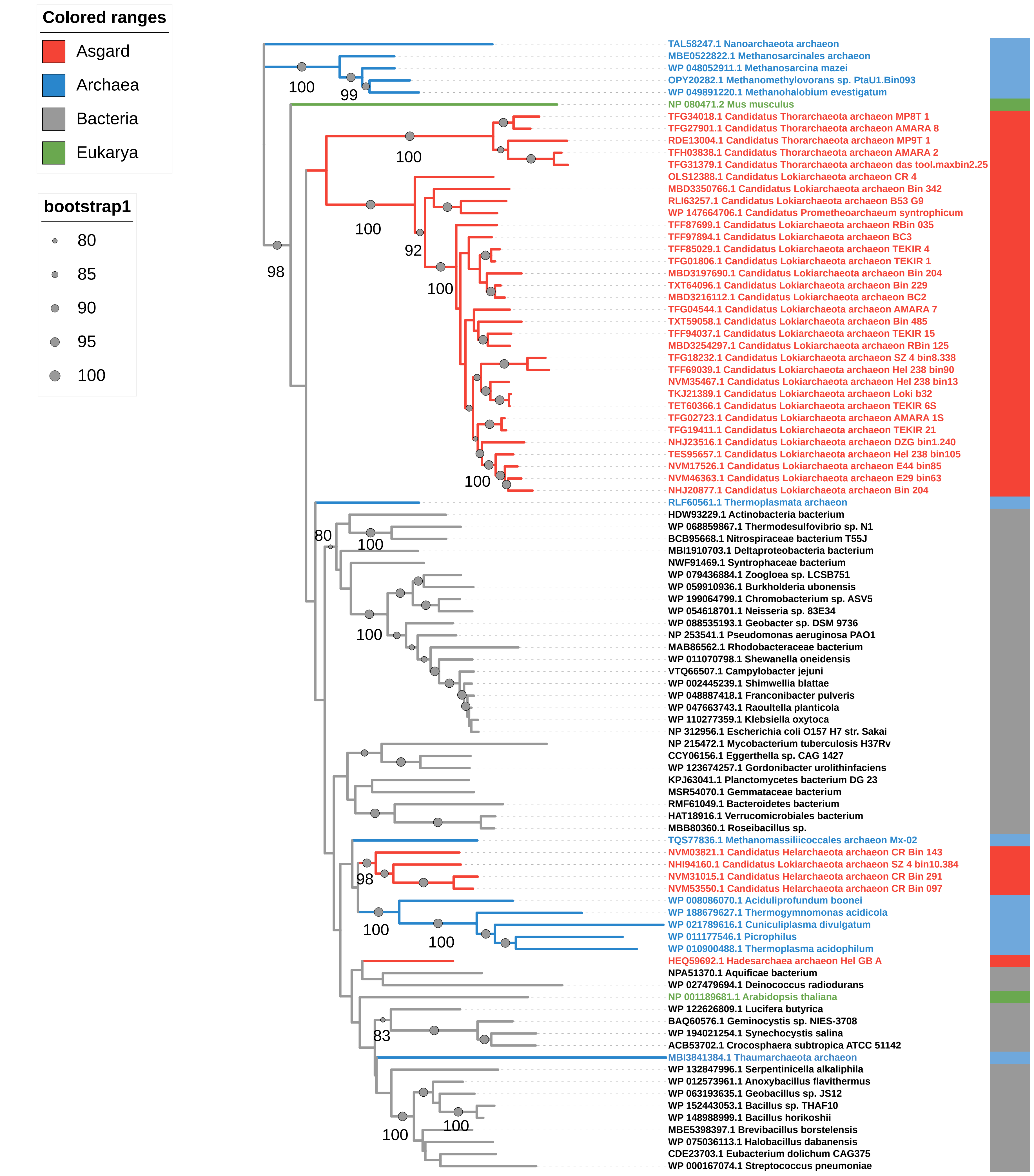

Supplementary Figure 9: Non-rooted IQ-TREE of PurH with bootstrap values.

Supplementary Figure 10: Non-rooted IQ-TREE of MTHFS with bootstrap values.

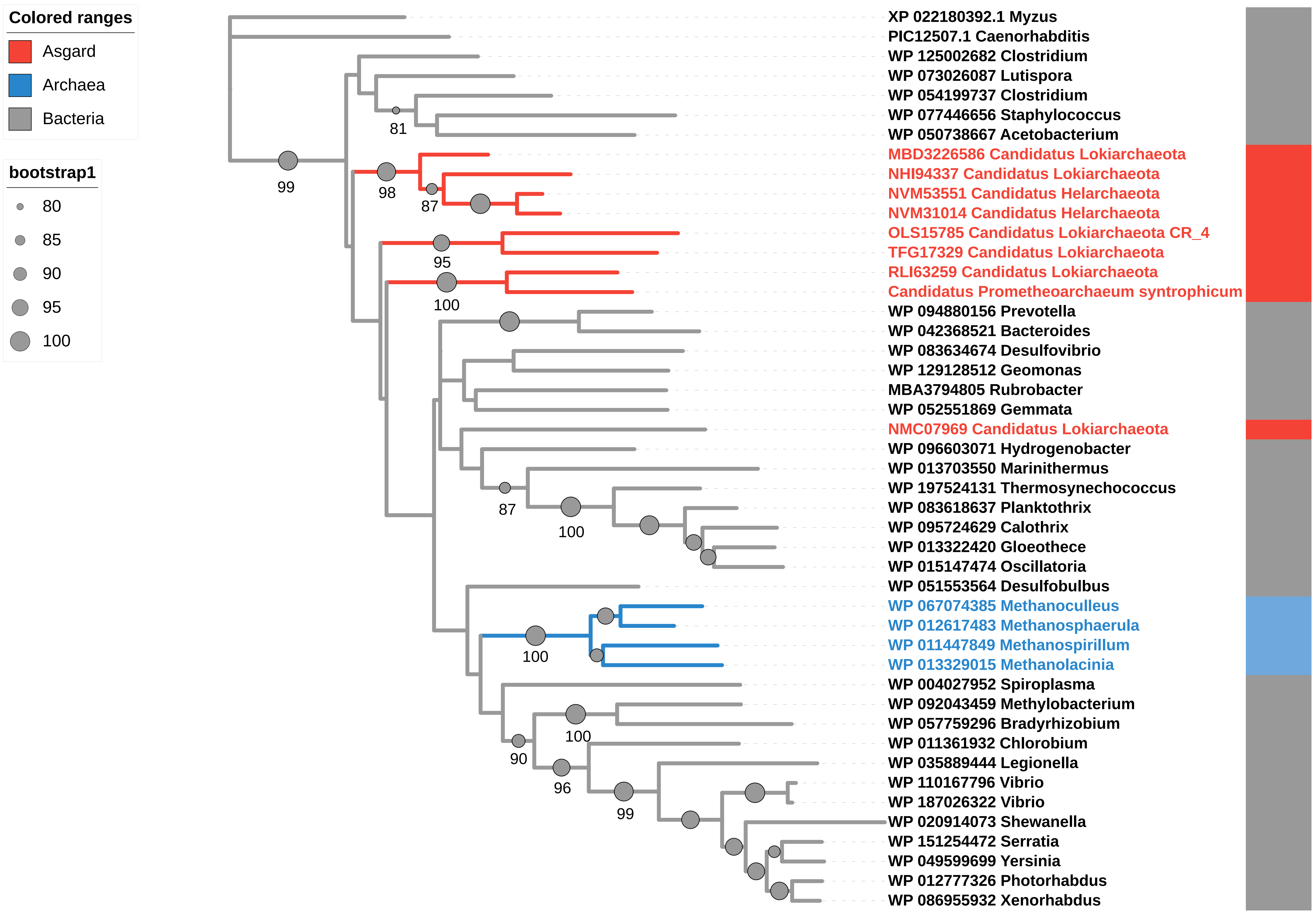

Supplementary Figure 11: Non-rooted IQ-TREE of MTHFR with bootstrap values.

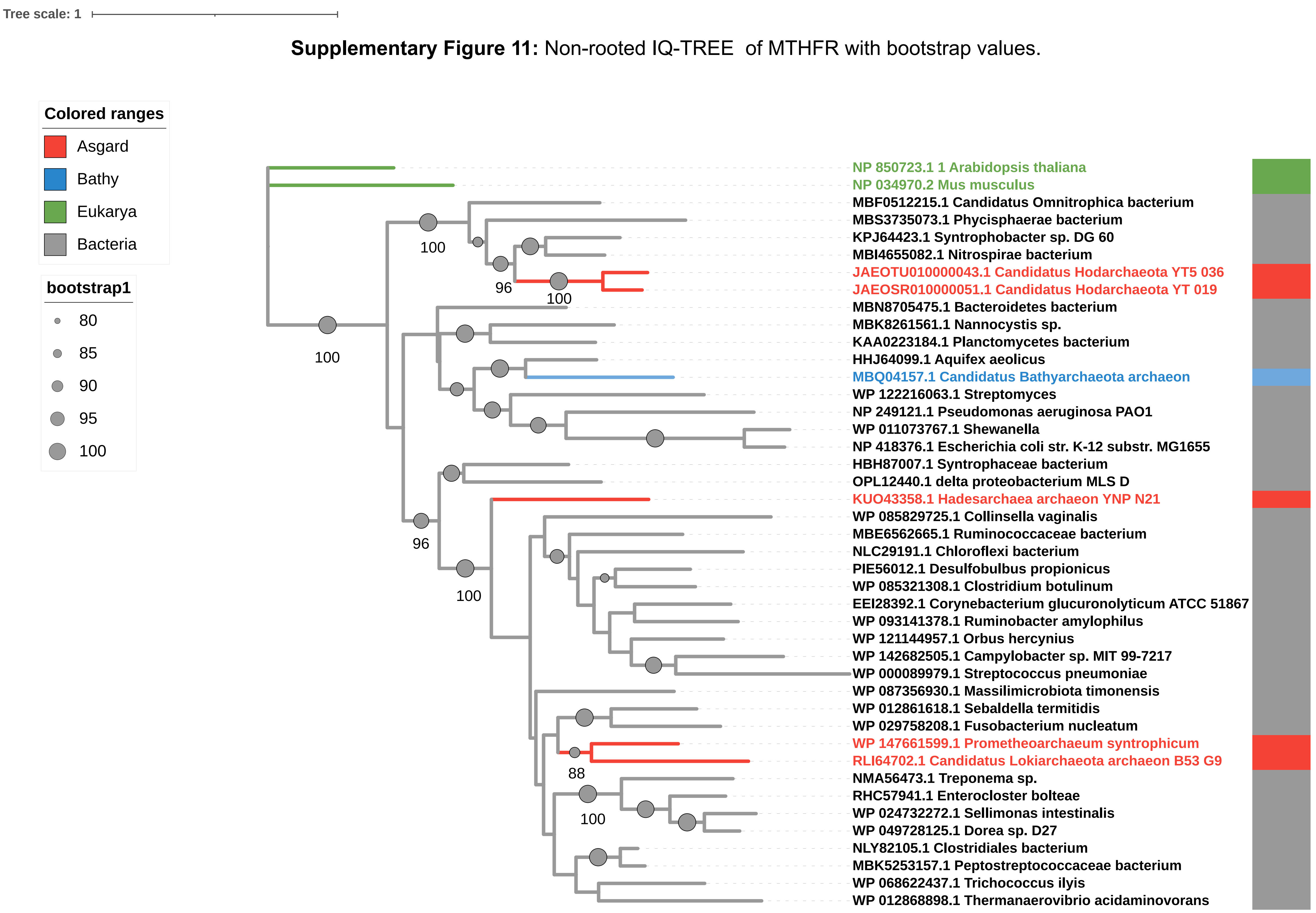

Tree scale: 1

Supplementary Figure 12: Non-rooted IQ-TREE of MetH with bootstrap values.

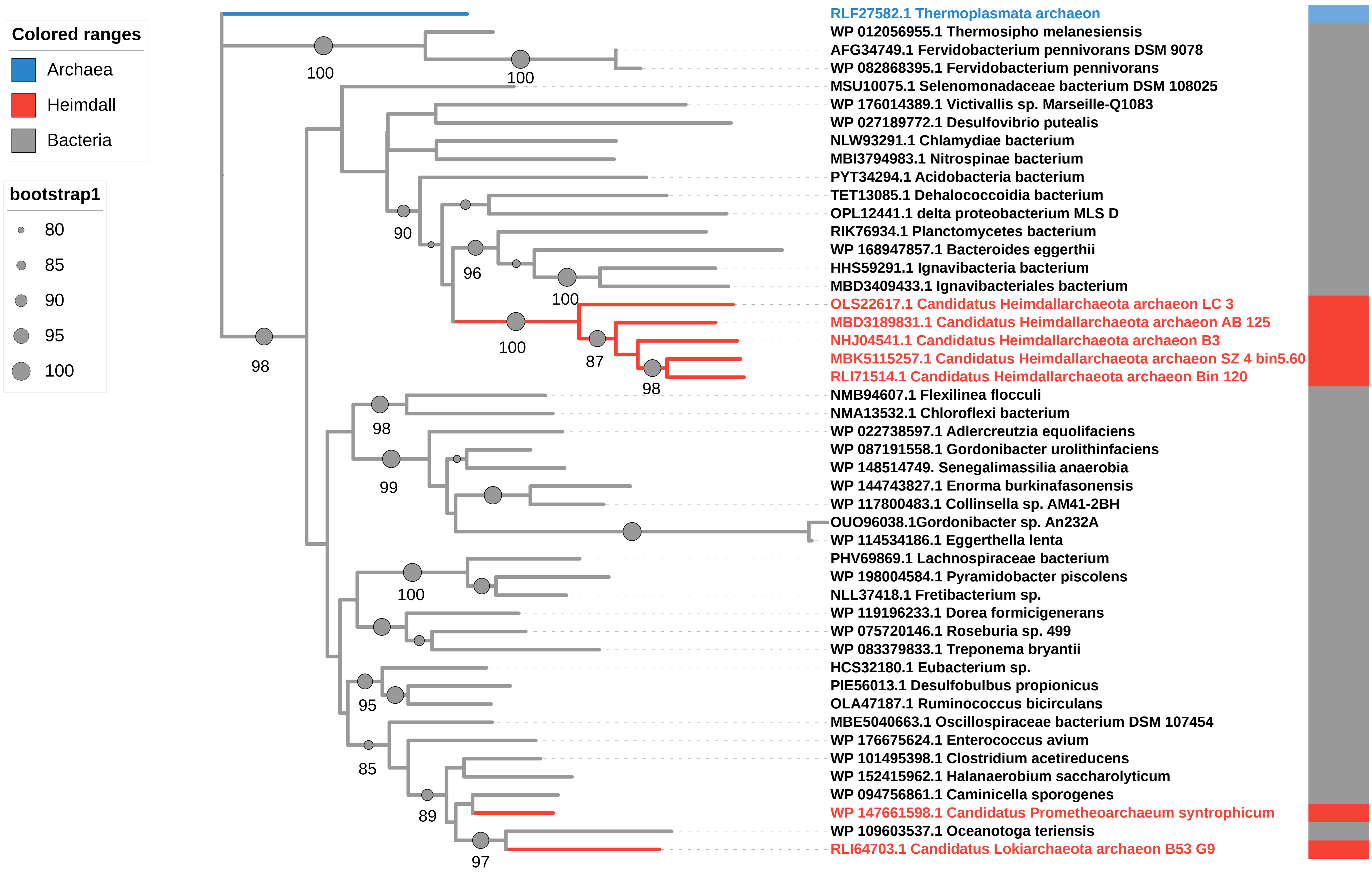

Supplementary Figure 13: Non-rooted IQ-TREE of FolD with bootstrap values.

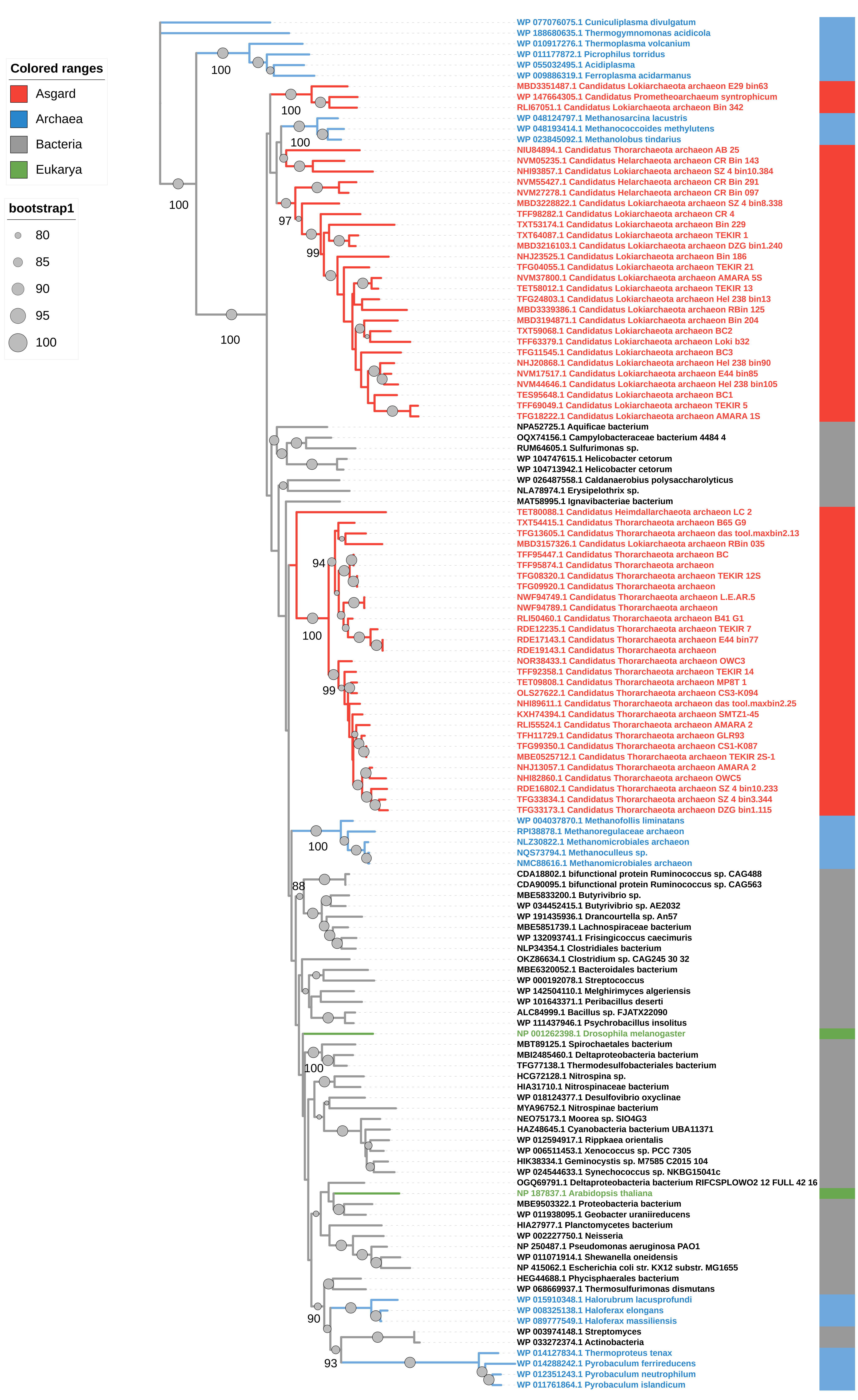

Supplementary Figure 14:

ThyX reconciliation analysis

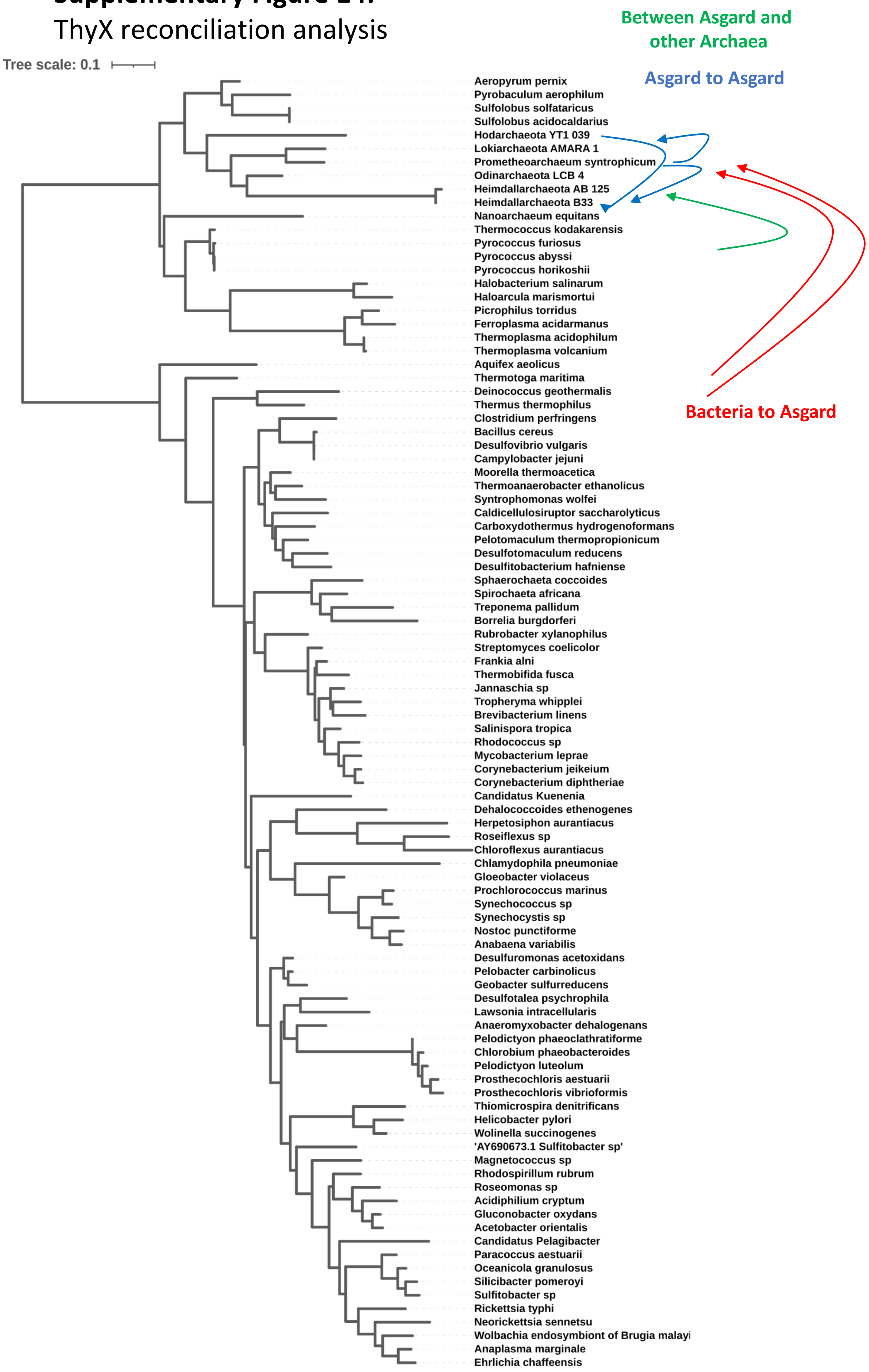

Supplementary Figure 15: ThyA reconciliation analysis

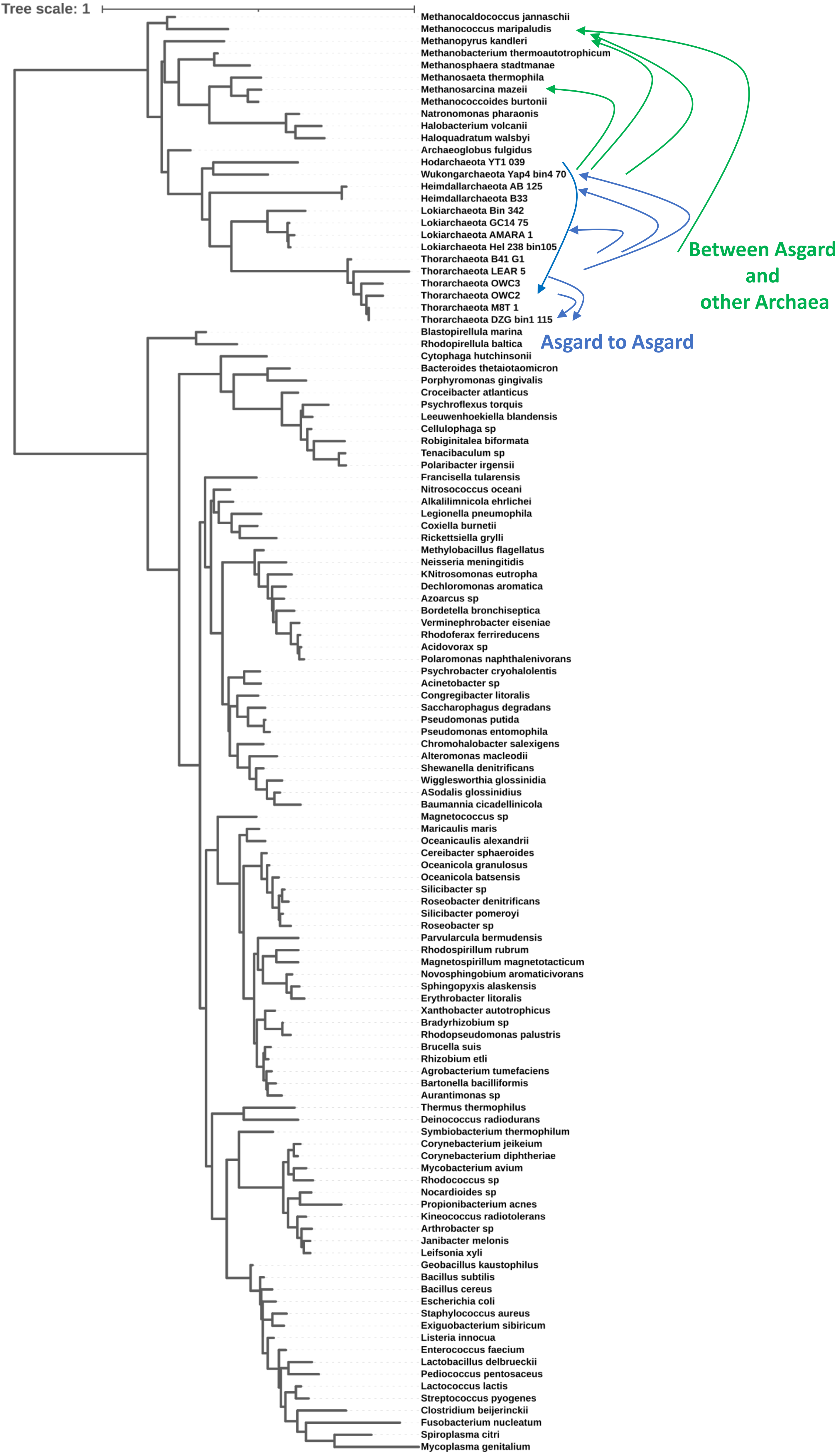

Supplement: Supplementary file 1 — Supplementary Information [file 41467_2023_36487_MOESM1_ESM.pdf]
