# Supplementary material for: Bacterial origins of thymidylate metabolism in Asgard archaea and Eukarya
Source: Nat Commun. 2023 Feb 15;14:838. doi: 10.1038/s41467-023-36487-z (PMC9931769; doi:10.1038/s41467-023-36487-z)
Supplement: Supplementary file 3 — Description of Additional Supplementary Files [file 41467_2023_36487_MOESM3_ESM.pdf]

Description of Additional Supplementary Files:

Supplementary Data 1: Distribution of folate-dependent enzymes in Asgard archaea and predicted HGT events using HGTector.

Source Data: Raw data for activity measurements (Fig. 7).
